# The essence of governance in health development

**DOI:** 10.1186/1755-7682-4-11

**Published:** 2011-03-28

**Authors:** Joses Muthuri Kirigia, Doris Gatwiri Kirigia

**Affiliations:** 1 World Health Organization, Regional Office for Africa, B.P. 06, Brazzaville, Congo; 2 Centres for Disease Control/WHO Consultant, P.O. Box 529, Freetown, Sierra Leone

## Abstract

**Background:**

Governance and leadership in health development are critically important for the achievement of the health Millennium Development Goals (MDGs) and other national health goals. Those two factors might explain why many countries in Africa are not on track to attain the health MDGs by 2015. This paper debates the meaning of 'governance in health development', reviews briefly existing governance frameworks, proposes a modified framework on health development governance (HDG), and develops a HDG index.

**Discussion:**

We argue that unlike 'leadership in health development', 'governance in health development' is the sole prerogative of the Government through the Ministry of Health, which can choose to delegate (but not abrogate) some of the governance tasks. The general governance domains of the UNDP and the World Bank are very pertinent but not sufficient for assessment of health development governance. The WHO six domains of governance do not include effective external partnerships for health, equity in health development, efficiency in resource allocation and use, ethical practises in health research and service provision, and macroeconomic and political stability. The framework for assessing health systems governance developed by Siddiqi *et al *also does not include macroeconomic and political stability as a separate principle. The Siddiqi *et al *framework does not propose a way of scoring the various governance domains to facilitate aggregation, inter-country comparisons and health development governance tracking over time.

This paper argues for a broader health development governance framework because other sectors that assure human rights to education, employment, food, housing, political participation, and security combined have greater impact on health development than the health systems. It also suggests some amendments to Siddigi *et al*'s framework to make it more relevant to the broader concept of 'governance in health development' and to the WHO African Region context.

**Summary:**

A strong case for broader health development governance framework has been made. A health development governance index with 10 functions and 42 sub-functions has been proposed to facilitate inter-country comparisons. Potential sources of data for estimating HDGI have been suggested. The Governance indices for individual sub-functions can aid policy-makers to establish the sources of weak health governance and subsequently develop appropriate interventions for ameliorating the situation.

## Background

An Editorial in the African Journal of Health Sciences delved into the 'The essence of leadership in health development' [1]. However, there is often confusion between the terms 'leadership' and 'governance'. The Editorial argues that the Ministry of Health Headquarters, provincial medical officers of health, district medical officers of health, and officers-in-charge of health facilities are all leaders but they do not have the monopoly of leadership in health development. It concludes that all health workers and parents play public health leadership roles and their effectiveness could be enhanced through empowerment with appropriate leadership skills training. This paper debates on what governance in health development entails. It argues that unlike 'leadership in health development', 'governance in health development' is the sole prerogative of the Government through the Ministry of Health, which can choose to delegate (but not abrogate) some of the governance tasks.

The World Health Report 2000 [2] delineated four functions of national health systems: stewardship, health financing, resource (input) creation and health services provision. The report uses terms 'stewardship' and 'governance' interchangeably. HarperCollins [3] dictionary defines a steward as a "person who administers another's property" (p.620). Since the Ministry of Health administers the public health system, it is a steward. Faith-based Organizations Health Associations, in their stewardship role, also oversees the running of church provided health services. The Board's of Directors of private-for-profit hospitals, in their stewardship role, oversees the operations of private hospitals on half of shareholders. This implies that the Ministry of Health does not have monopoly over stewardship of a national health system.

Unlike stewardship, governance is the sole prerogative of a national government, i.e. the executive policy-making body that exercises political authority over a country. A government through its system(s) rules or governs a country. HarperCollins [3] dictionary defines the word govern as "rule, administer, command, control (curb), direct, guide, handle, lead, manage, order, restrain, check, discipline, master, regulate, subdue, tame" (p.285). Thus, the scope of the governance role of government extends far beyond its stewardship role.

## Discussion

### Overview of existing governance frameworks

The United Nations Development Programme (UNDP) five principles of good governance include: legitimacy and voice (participation and consensus orientation), direction (strategic vision), performance (responsiveness, and effectiveness and efficiency), accountability (and transparency), and fairness (equity and inclusiveness, and rule of law) [4].

The World Bank's three clusters (with six domains) of governance include: processes by which those in authority are selected and replaced (voice and accountability, and political instability and violence); ability of government to formulate and implement sound policies (government effectiveness and regulatory burden); and respect of citizens and the state for institutions which govern their interaction (rule of law and control of corruption) [5].

The World Health Report 2000 [2] six domains of stewardship include: generation of intelligence, formulating strategic policy framework, ensuring tools for implementation (powers, incentives, and sanctions), building coalitions/partnerships, ensuring fit between policy objectives and organizational structure and culture, and ensuring accountability.

The slightly amended World Health Organization [6] health systems framework consists of six building blocks: service delivery; health workforce; information; medical products, vaccines and technologies; financing; and leadership/governance. The latter building block has six functions: policy guidance, intelligence and oversight, collaboration and coalition building, regulation, and accountability.

Siddiqi *et al *[7] framework for assessing governance of the health system has ten principles (and 22 domains): strategic vision (long vision, comprehensive development strategy including health), participation and consensus orientation (participation in decision-making process, stakeholder identification and voice), rule of law (legislative process, interpretation of legislation to regulation and policy, enforcement of laws and regulations), transparency (transparency in decision making and resource allocation), responsiveness of institutions (response to population needs and to regional local health needs), equity and inclusiveness (equity in access to care, fair financing of health care, disparities in health), effectiveness and efficiency (quality of human resources, communication processes, capacity for implementation), accountability (internal and external accountability), intelligence and information (information generation, collection, analysis and dissemination), and ethics (principles of bioethics, health care and research ethics). The framework assesses each of the 22 domains along three levels (national, health policy formulation, and policy implementation) and 63 questions (5 context related, 25 descriptive, 27 analytical/process related, and 6 outcomes related). Siddiqi *et al *[7] framework is the most comprehensive framework for assessing governance of health systems to date.

The governance domains of the UNDP and the World Bank are very pertinent but not sufficient for assessment of health development governance. That is understandable since they were developed for assessing general governance. The WHO six domains of governance do not include effective external partnerships for health, equity in health development, efficiency in resource allocation and use, ethical practises in health research and service provision, and macroeconomic and political stability. Siddiqi *et al *[7] also does not include macroeconomic and political stability as a separate principle, which is understandable because their framework is for assessing health systems governance.

In this paper we are arguing for a broader health development governance framework. Why? This is because of the fact that other sectors that assure human rights to education, employment, food, housing, political participation, and security combined have greater impact on health development than the health systems. For example, the significant negative impact of political and macroeconomic instability on health development has been starkly demonstrated in the diminished health indicators of the African countries that have undergone various forms of political and macroeconomic turmoil.

Siddiqi *et al *[7] does not propose a way of scoring the various domains to facilitate aggregation, inter-country comparisons and health development governance tracking over time. The following section suggests some amendments to Siddigi *et al*'s framework to make it more relevant to the WHO African Region context.

### Modified Framework for Health Development Governance

Table 1 presents a modified framework for assessing health development governance. This framework has ten functions and forty-two sub-functions of governance. First, public health leadership and management, which has five sub-functions, i.e. leadership responsibilities [8,9], national health policy (NHP) [10], national health strategic plan (NHSP) [10], dissemination of NHP and NHSP, and implementation of NHSP [11]. Siddigi *et al *[7] refers to this function as strategic vision. Second, rule of health-related laws function contains two sub-functions, i.e. existence of health-related legislations and their enforcement.

**Table 1:** Modified framework for assessing health development governance

Functions of health development governance	On a scale of 0% to 100%, assess the performance of each of the following sub-functions of health development governance:
1. Public health leadership and management	***1.1 Leadership responsibilities: ***Extent to which the Ministry of Health gives direction and effectively communicates that vision to align people with it; protect the health system from external threats; clarifies the roles and responsibilities of various actors; manages conflict internally and externally; motivates and inspires health workers (and other stakeholders) by satisfying their basic human needs to sustain their focus on the health development vision; and shapes the norms (including challenges unproductive norms) [8,9]. ***1.2 National health policy (NHP): ***existence of an updated national health policy based on a thorough situation analysis of health systems goals (health, fairness in financing and responsiveness to non-medical expectations) and functions (governance, health financing, resource creation and health service provision) and policy dialogue, and existence of clearly spelt out strategic vision for health development, guiding principles and underlying values, goals, health development priorities (based on rational criteria, e.g. cost-effectiveness analysis), implementation framework, resource mobilization mechanisms, and modalities for monitoring and evaluation [10]. ***1.3 National health strategic plan (NHSP): ***contain a background; situation analysis (socioeconomic context; health situation; state of health services supply and demand; strengths, weaknesses, opportunities and threats); strategic health development priorities (vision, mission, goal, guiding principles, objectives, targets, strategic thrusts, expected results/outcomes, activities and performance indicators); resource requirements, including human resources, building space, vehicles, equipment, materials and supplies, information, communication and technology (ICT); finance plan (containing prospective estimates cost, available funds, financing gap and ways of bridging the gap); implementation framework specifying the roles and responsibilities of various people, institutions and organizations involved in health development; monitoring and evaluation, including mechanisms, schedule and cost; conclusion; and appendices [10]. ***1.4 Dissemination of NHP and NHSP: ***the NHP and NHSP are widely available at national, provincial/regional, district and community levels in relevant local languages. ***1.5 Implementation: ***Extent to which NHSP has been translated into results-oriented operational programmes and plans as expressed in medium-term expenditure frameworks and annual programme budgets [11].
2. Rule of health-related laws	***2.1 Existence of health-related legislations: ***Existence of public health laws related to governance, health financing, resource/input creation (essential health technologies, human resources, and infrastructure), provision of personal and public health services, research for health, ethical practises [7]. ***2.2 Enforcement of health-related legislations: ***the extent to which various health-related laws are applied at all levels of health system (and government) to administer governance, health financing, resource/input creation (essential health technologies, human resources, infrastructure), provision of personal and public health services, research for health, ethical practises [7].
3. Community participation & responsiveness	***3.1 Participation in NHP and NHSP development: ***Extent to which communities (either directly or through elected leaders) are involved in the health needs assessment, national health policy development, and planning of health development [12,13]. ***3.2 Participation in NHSP implementation: ***Extent to which communities (either directly or through elected leaders) are involved in management of health services and other health enhancing services (e.g. water, sanitation, environmental pollution control). ***3.3 Participation in tracking of progress: ***Extent to which communities (either directly or through elected leaders) are involved in monitoring and evaluation in the achievement of health development objectives and targets spelt out in the NHSP. ***3.4 Responsiveness to communities non-medical expectations: ***Extent to which health systems exercise respect for persons (dignity, autonomy in choice of interventions and confidentiality) and are client-oriented (prompt, adequate basic amenities, access to social support networks, choice of provider) [14].
4. Effective internal and external partnerships for health	***4.1 Intersectoral action: ***Existence of vibrant intersectoral committees for tracing progress on socioeconomic determinants of health [15,16]. ***4.2 Public-private partnerships: ***Extent to which the legislative and policy environment forges partnerships with the faith-based organizations and private-for-profit sector in health financing, health systems input creation and health services provision to facilitate implementation of NHP and NHSP [17]. ***4.3 Alignment of aid flows to national health development priorities: ***(i) Percentage of aid flows for health development channelled through general government budget support [11]. ***4.4 Strengthen capacity by coordinated support: ***Percentage of technical cooperation flows implemented through coordinated programmes consistent with NHSP [11]. ***4.5 Use of country procurement and public financial management systems: ***Percentage of donor aid that flow through recipient/partner country procurement and public financial management systems [11]. ***4.6 Strengthen national capacity by avoiding parallel implementation structures: ***number of parallel health project implementation units in a country [11]. ***4.7 Aid is more predictable: ***Percent of health-related aid disbursed according to multi-year frameworks [11]. ***4.8 Aid is untied: ***Percentage of bilateral aid for health that is untied to donor conditionality [11]. ***4.9 Shared analysis: ***Percentage of health-related (a) field missions and/or (b) country analytic work undertaken jointly between the cluster of health donors and national government [11]. ***4.10 Sufficient integration of global programmes and initiatives into NHSP: ***Percentage of global programmes (e.g. Global Fund for Aids, Tuberculosis and malaria; GAVI) and initiatives supporting the implementation of NHSP [11].
5. Horizontal and vertical equity in health systems	***5.1 Horizontal equity: ***Extent to which there is the allocation of equivalent resources for people with equivalent capacity to benefit from health enhancing health interventions and socio-economic interventions [16,18-20]. ***5.2 Vertical equity: ***Extent to which there is allocation of different resources for people with different levels of capacity to benefit from health enhancing health interventions and socio-economic interventions [16,18-20]. ***5.3 Health fairness in financial contribution (HFC): ***Extent to which the ratio of total contribution to health from each household through all payments mechanisms (HE) to that household's capacity to pay (CTP) - which is the effective non-subsistence income - is identical for all households, independent of the household's health status or use of the health system [19], i.e. *HFC *= *HE/CTP*.
6. Efficiency in resource allocation and use	***6.1 Allocative efficiency: ***Percentage of various levels of fixed health facilities allocating health resources to their most highly valued uses [21]. ***6.2 Technical efficiency: ***Percentage of various levels of fixed health facilities using physical health systems inputs to produce either health services without waste [22-27]. ***6.3 Productivity growth: ***Percentage of various levels of fixed health facilities experiencing total factor productivity growth due to efficiency improvement and/or technological growth [28-30]. ***6.4 Institutionalization of efficiency monitoring: ***Extent to which economic efficiency monitoring has been institutionalized within the national health management information system [23].
7. Accountability and transparency in health development	***7.1 Existence of transparent results-oriented reporting and assessment frameworks ***to assess progress against NHSP targets indicators [11]. ***7.2 Diagnostic reviews: ***Extent to which diagnostic reviews of national arrangements and procedures for public financial management, accounting, auditing, procurement, results frameworks and monitoring provide reliable assessments of performance, transparency and accountability of country systems [11]. ***7.3 Use of information from diagnostic reviews: ***Extent to which evidence from diagnostic reviews is used in the design of reforms to ensure that national systems, institutions and procedures for managing all health resources are effective, accountable and transparent [11]. ***7.4 Publishing of audit reports for public consumption: ***Extent to which reliable and timely budget execution and audit reports are transparently reviewed by relevant parliamentary committees and published in mass media for public scrutiny [11].
8. Evidence-based decision-making	***8.1 National health research systems: ***Existence of a health research policy and strategic plans that are being implemented as evidenced in research outputs and their use in health policy, planning and decision-making [31,32]. ***8.2 Health knowledge management systems (HKMS): ***Existence of a functional HKMS that does acquisition, creation (probably through research and practise), diffusion, application and evaluation/improvement of knowledge [33]. ***8.3 Health management information systems: ***Extent to which a country has legal and policy frameworks supported by sufficient human resources, financing and infrastructure; core health indicators identified covering determinants of health, health system inputs, outputs and outcomes; key data available from six main sources and standards for their use - for census, vital events monitoring, health facilities statistics, public health surveillance, population-based surveys and resource tracking; optimal processes for collecting, sharing and storing data, data flows and feedback loops; dissemination of information and effective use of data for policy and advocacy, planning and priority setting, resource allocation, and implementation and action [34]. ***8.4 Information, Communication and Technology Connectivity: ***(i) Existence of a comprehensive national policy and a legal and strategic framework to guide and nurture the growth of ICT, while at the same time protecting the welfare of its citizens. (ii) Extent to which the necessary investment in ICT infrastructure, including fixed phone lines installation, equipment (e.g. computers, servers, networks) and Internet connectivity in the entire health system, i.e. from the Ministry of Health headquarters down to the level of community-based public health programmes [35].
9. Ethical practises in health research and service provision	***9.1 International ethical guidelines for medical practice and health research: ***Extent to which a country have adapted appropriately international ethical guidelines for medical practice (e.g., the International Code of Medical Ethics of the World Medical Association or the International Conference on Harmonization guidelines for Good Clinical Practice) and biomedical research involving human subjects, made them available to all national health and health-related research institutions and health facilities, and are being adhered to [36]. ***9.2 Bioethics review system: ***Existence of operational bioethics research review system, which includes national, regional, district and institutional (health facility) ethics committees for protecting the dignity, integrity and health safety of all its citizens participating in research and those consuming health services [37]. ***9.4 Institutionalization of ethics training: ***Extent to which a country has institutionalized training in ethics and human rights in relation to health at all stages of the education and training of all health workers, including medical, public health and nursing schools [37,38].
10. Macroeconomic and political stability	***10.1 Link between national economic development plan (NEDP), Poverty Reduction Strategy Paper (PRSP) and NHP/NHSP: ***Existence of NEDP and PRSP with a health component linked with the NHP and NHSP [39]. ***10.2 Existence of a medium-term expenditure framework (MTEF): ***Existence of a MTEF with a clear health component [39]. ***10.3 Political stability: ***Existence of non-violent processes by which those in authority are selected and replaced [40,41].

Third, community participation and responsiveness function has four sub-functions, i.e. participation in NHP and NHSP development process, participation in NHSP implementation, participation in tracking progress in implementation of NHSP, and responsiveness to community's legitimate non-medical expectations [12-14]. Siddiqi *et al *[7] principles of responsiveness of institutions and participation and consensus orientation are merged into one function of community participation and responsiveness. By assuring community participation in the planning, management and monitoring of health services, the institutions are partially being responsive.

Fourth, effective internal and external partnerships for health function has ten sub-functions, namely: inter-sectoral action [15,16], public-private partnerships [17], alignment of aid flows to national health development priorities, strengthening capacity by coordinated support, use of country procurement and public financial management systems, strengthening national capacity by avoiding parallel implementation structures, more predictable aid, untied aid, shared analysis, and sufficient integration of global programmes and initiatives into NHSP [11]. The need for inter-sectoral action with agriculture, animal husbandry, communications, education, employment, industry, food, housing, political participation, public works, security and transport sectors is critical for addressing all determinants of health development [15,16]. Governments also have an important role in mobilizing, nurturing, coordinating and managing external support to maximize its impact on health development.

Five, horizontal and vertical equity in health systems function has three sub-functions, i.e. horizontal equity in access of health services [18-20], vertical equity in access of health services [16,18-20], and fairness in financial contributions [18,19]. Six, efficiency in resource allocation and use function has four sub-functions, i.e. allocative efficiency [21], technical efficiency [22-27], productivity growth [28-30], and institutionalization of efficiency monitoring [23]. Seven, accountability and transparency in health development function has four sub-functions, i.e. existence of transparent results-oriented reporting and assessment frameworks; diagnostic reviews; use of information from diagnostic reviews; and publishing of audit reports for public consumption [11].

Eight, evidence-based decision-making function has four sub-functions, i.e. national health research systems [31,32]; health knowledge management systems [33]; health management information systems [34]; publishing of audit reports for public consumption; and information, communication and technology connectivity [35]. Nine, ethical practises in health research and service provision function, which has three sub-functions, i.e. international ethical guidelines for medical practice and health research, bioethics review system, and institutionalization of ethics training in all schools of medicine, nursing, public health and allied health sciences [36]. This function is critically important to assure adherence to the international ethical principles of beneficence, non-malfeasance, autonomy, justice, dignity, truthfulness and honesty [37] even in settings, like Africa, where principal-agency relation in health-related biotechnology transfer is rather weak [38].

Ten, macro-economic and political stability function is divided into three sub-functions, i.e. link between national economic development plan (NEDP) [39], Poverty Reduction Strategy Paper (PRSP) and NHP/NHSP, existence of a medium-term expenditure framework (MTEF), and political stability [40,41].

### Health Development Governance Index

**Figure 1:**
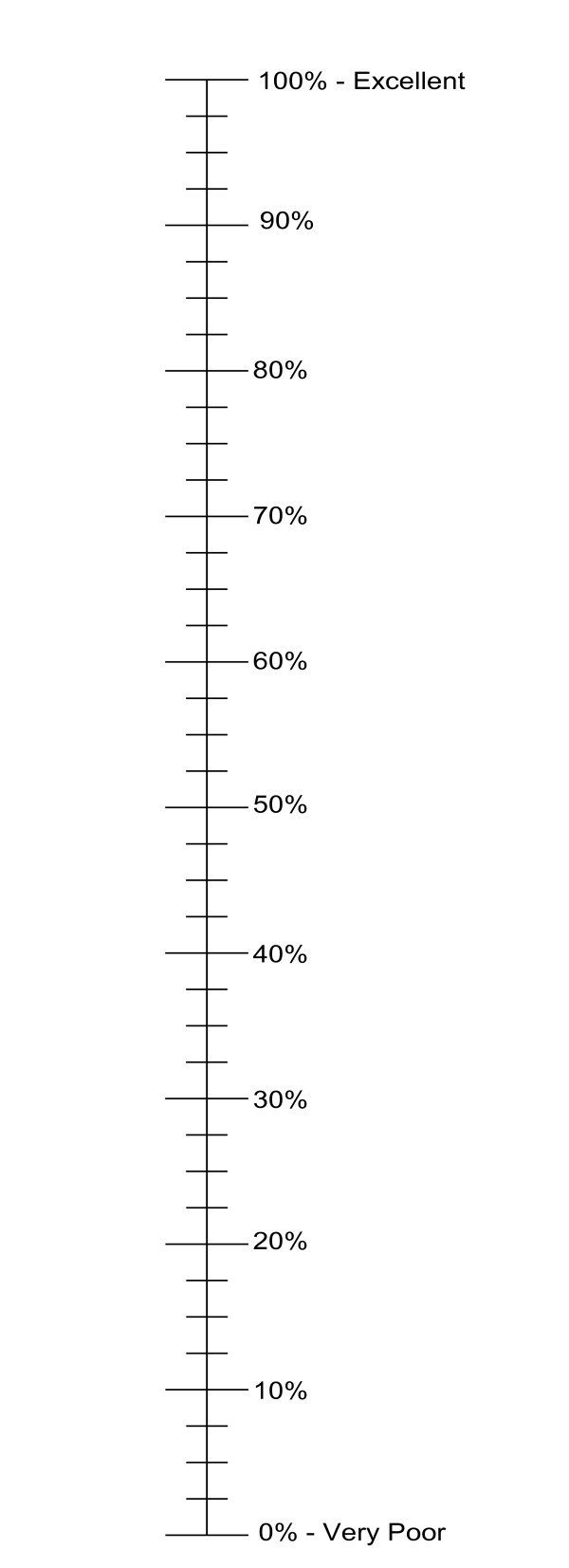
**Health development governance thermometer scale**.

The health development governance (HDG) framework discussed above has 10 functions and 42 sub-functions. Each of the functions can be measured using a governance thermometer scale of 0% (very poor) to 100% (excellent) (Figure 1). The scale is authors' own construction. The Health Development Governance Index (HDGI) is the arithmetic mean of 42 indices, namely the index of each of the sub-functions in Table 2. All the indices are computed using the following general formula:

**Table 2:** An hypothetical country health development governance index

*Sub-functions of health development governance*	*Actual* *Score* *(A)*	*Maximum* *(B)*	*Minimum* *(C)*	*Sub-function* *Governance* *Index* *(D) = (A-C)/(B-C)*
1.1 Leadership responsibilities	20	100	0	0.20
1.2 National health policy (NHP)	15	100	0	0.15
1.3 National health strategic plan (NHSP)	10	100	0	0.10
1.4 Dissemination of NHP and NHSP	40	100	0	0.40
1.5 Implementation of NHSP	32	100	0	0.32
2.1 Existence of health-related legislation	32	100	0	0.32
2.2 Enforcement of health-related legislations	23	100	0	0.23
3.1 Participation in NHP and NHSP development	12	100	0	0.12
3.2 Participation in NHSP implementation	13	100	0	0.13
3.3 Participation in tracking of progress	11	100	0	0.11
3.4 Responsiveness to communities non-medical expectations	14	100	0	0.14
4.1 Intersectoral action	15	100	0	0.15
4.2 Public-private partnerships	16	100	0	0.16
4.3 Alignment of aid flows to national health development priorities	17	100	0	0.17
4.4 Strengthen capacity by coordinated support	18	100	0	0.18
4.5 Use of country procurement and public financial management systems	19	100	0	0.19
4.6 Strengthen national capacity by avoiding parallel implementation structures	20	100	0	0.20
4.7 Aid is more predictable	21	100	0	0.21
4.8 Aid is untied	32	100	0	0.32
4.9 Shared analysis	33	100	0	0.33
4.10 Sufficient integration of global programmes and initiatives into NHSP	43	100	0	0.43
5.1 Horizontal equity	54	100	0	0.54
5.2 Vertical equity	65	100	0	0.65
5.3 Fairness in financial contribution	55	100	0	0.55
6.1 Allocative efficiency	22	100	0	0.22
6.2 Technical efficiency	21	100	0	0.21
6.3 Productivity growth	25	100	0	0.25
6.4 Institutionalization of efficiency monitoring	51	100	0	0.51
7.1 Existence of transparent results-oriented reporting and assessment frameworks	52	100	0	0.52
7.2 Diagnostic reviews	53	100	0	0.53
7.3 Use of information from diagnostic reviews	54	100	0	0.54
7.4 Publishing of audit reports for public consumption	61	100	0	0.61
8.1 National health research systems	62	100	0	0.62
8.2 Health knowledge management systems	62	100	0	0.62
8.3 Health management information systems	63	100	0	0.63
8.4 Information, Communication and Technology Connectivity	64	100	0	0.64
9.1 International ethical guidelines for medical practice and health research	65	100	0	0.65
9.2 Bioethics review system	71	100	0	0.71
9.4 Institutionalization of ethics training	72	100	0	0.72
10.1 Link between NEDP, PRSP and NHP/NHSP	15	100	0	0.15
10.2 Existence of a MTEF	20	100	0	0.20
10.3 Political stability	10	100	0	0.10
				14.73
**OVERALL GOVERNANCE INDEX:**				**0.35**

Where xi is the HDG indicator (sub-function) such as leadership responsibilities, existence of health-related legislation, inter-sectoral action, horizontal equity, allocative efficiency, existence of transparent results-oriented reporting and assessment frameworks, bioethics review system, national health research systems, etc.

For example, the Leadership Responsibilities index (LR) can be expressed as follows:

where LRI is the leadership responsibility index, Actual (LR) is the actual Leadership Responsibility score, Minimum (LR) is the minimum leadership responsibility score, and Maximum (LR) is the maximum leadership responsibility score. For example, assuming the global minimum leadership responsibility score and the global maximum responsibility scores are set equal to 0% and 100% respectively and the actual leadership responsibility score for a hypothetical country in Table 1 is 20%, the LRI can be obtained as follows:

The indices for each of the remaining 41 sub-functions can be calculated in a similar manner. And once individual sub-functions indices have been obtained, the overall Health Development Governance Index (HDGI) can be obtained using the following formula:

where  is summation from sub-function 1 index to sub-function 42 index; HDGSFI is the health development governance sub-function index; N is the total number of sub-functions in the assessment framework. In the hypothetical example given in Table 2 the HDGI has been obtained as follows:

Since governance is measured on a scale of 0 (or 0%) to 1 (or 100%), the above HDGI of 0.35 implies that the health development governance in this hypothetical country is below average. If instead, the hypothetical country's HDGI was 50%, it would have signified average health development governance. The above formula is similar to that used by the United Nations Development Programme in calculating the Human Development Index [42]. In reality, the scoring (using the thermometer scale) of the different sub-functions can be done by geographically representative national committees of informed persons.

The Governance indices for individual sub-functions can aid policy-makers to locate the sources of poor governance and then to develop appropriate interventions for ameliorating the situation. Those indices could be conducted every two or three years among all countries in the WHO African Region. Therefore, every two or three years, the WHO Regional Committee Ministers of Health from the African Region can have peer review. Since Regional Committee meets every year, there would be no additional cost for organizing the peer review sessions. Such peer review mechanisms would motivate countries to improve health development governance and also share good practises.

### Possible sources of data for estimating HDGI

Countries that choose to estimate the HDGI may need to set up a national multi-disciplinary stakeholder Technical Working Group (TWG) to assess the current status of HDG. In addition, they might consider establishing a Steering Committee (SC) to oversee and facilitate the work. The TWG ought to be made up of appropriately qualified staff from all relevant sectors and programmes that deal with the ten functional domains of HDG. It is critically important for all relevant government sectors (especially those addressing various health determinants), health development partners, civil society organizations, and private health sector to be represented in both the TWG and SC. Wide participation will ensure that the results will be used to improve national HDG.

Table 3 shows the possible sources of data needed to assess the performance of each of the sub-functions of HDG. *Public health leadership and management: *Comprehensiveness of the NHP and NHSP can be assessed by reviewing the two documents against the WHO/AFRO guidelines [10]. The data on leadership responsibilities and dissemination of NHP and NHSP can be obtained through a survey of stakeholders, e.g. civil society, health workers (in both public and private sectors) and partners. The level of implementation of NHSP can be assessed through appraisal of annual health sector review reports and other monitoring and evaluation reports. Annual reviews are often based on NHIS data and some times complemented with routinely gathered primary data.

**Table 3:** Possible sources of data for computing national HDGI

*Sub-functions of health development governance*	*Possible data source*
1.1 Leadership responsibilities	Conduct a survey among samples of stakeholders, e.g. civil society, health workforce.
1.2 National health policy (NHP)	Review of NHP.
1.3 National health strategic plan (NHSP)	Review of NHSP.
1.4 Dissemination of NHP and NHSP	Conduct a survey among samples of public and private health sector managers at various levels of health system, e.g. headquarters, provinces/regions, and districts. The survey questionnaire should be administered to civil society organizations and partners.
1.5 Implementation of NHSP	Review of annual health sector review reports & other monitoring & evaluation reports. Plus review of national health information systems (NHIS) data.
2.1 Existence of health-related legislation	Review of existing health-related laws.
2.2 Enforcement of health-related legislations	Inclusion of relevant questions in the survey mentioned in 1.4 above.
3.1 Participation in NHP and NHSP development	Conduct a survey among parliamentarians and civic leaders or administrative leaders (especially chiefs).
3.2 Participation in NHSP implementation	Inclusion of relevant questions in the survey mentioned in 3.1 above.
3.3 Participation in tracking of progress	Inclusion of relevant questions in the survey mentioned in 3.1 above.
3.4 Responsiveness to communities non-medical expectations	Exit client surveys among samples of different levels of health facilities, e.g. tertiary, provincial/regional and district hospitals, and health centres.
4.1 Intersectoral action	In-depth interview with prime minister/president's office.
4.2 Public-private partnerships	Review of health-related legislation & interviews of leaders of faith-based and private-for-profit health service providers.
4.3 Alignment of aid flows to national health development priorities	Interviews with Ministry of Finance and health development partners.
4.4 Strengthen capacity by coordinated support	Interviews with Ministry of Health regarding existence of Sector-Wide Approaches, multi-donor steering committees or equivalent mechanisms.
4.5 Use of country procurement and public financial management systems	Review reports of the Public Expenditure and Financial Accountability (PEFA) initiative [44]. If the data does not already exist use PEFA framework [45] to conduct the assessment.
4.6 Strengthen national capacity by avoiding parallel implementation structures	
4.7 Aid is more predictable	
4.8 Aid is untied	
4.9 Shared analysis	
4.10 Sufficient integration of global programmes and initiatives into NHSP	Interviews with Ministry of Health, Ministry of Finance, GFATM and GAVI.
5.1 Horizontal equity	Analysis of household surveys, e.g., World Health Surveys [43], LSMS [49], DHS [50], and MICS [51]. Other sources include household budget surveys, census data, facility-based surveys (exit polls), and routine data from NHIS, vital registration, etc [48].
5.2 Vertical equity	
5.3 Fairness in financial contribution	
6.1 Allocative efficiency	National NHIS: (i) health facility service data, e.g. numbers of curative and preventive outpatient visits, numbers of hospital admissions and discharges, numbers of hospital deaths, numbers of diagnostic services, volume of community-based health services; (ii) quantities and values of resources, e.g. supplies, health workforce, finances, infrastructure. If data is not available centrally, there may be need to collect it from health facilities, using existing questionnaires [63,64].
6.2 Technical efficiency	
6.3 Productivity growth	
6.4 Institutionalization of efficiency monitoring	
7.1 Existence of transparent results-oriented reporting and assessment frameworks	This data should be obtained simultaneously with that in 4.5-4.9.
7.2 Diagnostic reviews	
7.3 Use of information from diagnostic reviews	
7.4 Publishing of audit reports for public consumption	
8.1 National health research systems (HRS)	Review existing Health Research Systems Analysis (HRSA) reports; and where non existent conduct an assessment of HRS using HRSA toolkit [65].
8.2 Health knowledge management systems (HKMS)	Review existing HKMS reports; and where they do not exist undertake an assessment of HKMS using "Research Matters" Knowledge Translation Toolkit [68].
8.3 Health management information systems	Review existing NHIS reports; and where non-existent conduct an assessment using Health Metrics Network tool [69].
8.4 Information, Communication and Technology Connectivity	Include relevant questions in the survey questionnaire mentioned in 1.4.
9.1 International ethical guidelines for medical practice and health research	This data should be collected simultaneously with that in 8.1 using same methods and tools.
9.2 Bioethics review system	
9.4 Institutionalization of ethics training	
10.1 Link between NEDP, PRSP and NHP/NHSP	Review of the NEDP, PRSP, NHP and NHSP
10.2 Existence of a MTEF	Review of MTEF document complemented with in-depth interviews with Ministry of Finance and Ministry of Health.
10.3 Political stability	Review of the national constitution, in-depth interview with chairperson of national legal bar association, and reference to both the Economist Intelligence Unit Democracy Index [40] and the Ibrahim Index [41].

#### Rule of law

Existence of health-related legislation can be assessed through review of existing health-related laws. The questions for assessing level of enforcement of health-related legislation may be included in the survey questionnaire for public health leadership and management.

#### Community participation and responsiveness

Firstly, data for use in assessing the level of community participation in formulation of NHP/NHSP and monitoring their implementation can be generated through administration of a questionnaire among parliamentarians and civic leaders or administrative leaders, e.g. chiefs. Secondly, responsiveness of health service providers to communities' non-medical expectations can be assessed through administration of responsiveness module of the World health survey questionnaire [43] among samples of clients exiting various levels of health facilities, e.g. tertiary, provincial/regional and district hospitals, and health centres.

#### Effective internal and external partnership for health

Firstly, data for assessing inter-sectoral action can be generated from in-depth interview with either prime minister's or president's office, depending on who chairs the cabinet. Secondly, review of health-related legislation and interviews with leaders of faith-based and private-for-profits health services providers can yield information on the extent to which legislative and policy environment fosters public-private partnerships.

Thirdly, interviews with Ministry of Finance and health development partners could yield information on percentage of aid flows for health development channelled through general government budget support. Fourthly, interviews with ministries of health would yield information on the existence of sector-wide approaches, multi-donor steering committees or equivalent donor coordination mechanisms.

Fifthly, the Public Expenditure and Financial Accountability (PEFA) initiative [44] reports contain information needed to assess extent of use of country procurement and public financial management systems, predictability of aid, whether aid is tied or not, and use of shared analyses. As at 7^th ^March 2011, about 30 WHO African region countries had reports on the PEFA Secretariat website. Where such information does not exist, there may be need to conduct assessment using the PEFA framework [45].

Information on whether there has been sufficient integration of global programmes and initiatives into NHSP can be obtained by conducting interviews with Directors of Policy and Planning at Ministry of Health, health sector focal persons in Ministries of Finance, and country representatives of GAVI and GFATM.

The data needed for accountability and transparency in health development should be collected together with that on effective international and external partnership.

#### Horizontal and vertical equity in health systems

Estimation of health inequality requires data on health variables (e.g.) and ordinal living standards measure [46]. Equity in utilization of health services or interventions entails data on utilization variables and ordinal living standards measure [47]. Benefit-incidence analysis calls for data on health service utilization variables, ordinal living standards measure, and unit subsidies [48]. Calculations of progressivity, catastrophic payments, or poverty impact of health financing require data on cardinal measure of living standards and user payments [48]. Analysis of equity in health systems is best done using data from household surveys. Since household surveys can be very expensive to conduct, it is advisable to use data from existing household survey datasets, e.g., Living Standards Measurement Study (LSMS) [49], Demographic and Health Surveys (DHS) [50], Multiple Indicator Cluster Surveys (MICS) [51] and World Health Surveys [43]. O'Donnell *et al *[48] is an excellent open access resource on how to analyze health equity using household survey data. The World Bank has also developed a free computer programme know as "ADePT-Health" for conducting health equity analyses [52].

#### Efficiency in resource allocation and use

Estimation of technical efficiency (TE) requires data on quantities of health system inputs, e.g. numbers (or time) of different cadres of health workforce, annual expenditure on pharmaceuticals, annual expenditure on non-pharmaceutical supplies, number of hospital beds; and volume of health service outputs, e.g. number of outpatient curative visits, outpatient preventive visits, community health outreach activities, inpatient admissions, inpatient discharges, and hospital deaths. In order to estimate allocative efficiency (AE), information on average inputs prices is needed in addition to data needed for TE. Calculation of productivity change requires all abovementioned input and output data for a number of time periods, e.g. a number of years. In the African region TE studies have been undertaken in Benin [53], Burkina Faso [54], Ethiopia [55], Ghana [22,56], Kenya [24,25], Namibia [57], Sierra Leone [23], South Africa [27,58,59] and Zambia [60]; AE studies have been conducted in Ghana [61] and Zambia [21]; and *Malmquist *total factor productivity analyses have been carried out in continental Africa national health systems [30], Angola [28], Botswana [62], Seychelles [29] and South Africa [26]. Therefore, results from such studies can be used in computation of HDGI. However, in countries where no such studies exist, it will be necessary to collect relevant data and do the efficiency and productivity change analyses. Data needed for efficiency analyses can be found in NHIS database. If the data is not centrally available in NHIS database, there may be need to collate it from health facilities, using existing questionnaires [63,64].

#### Evidence-based decision making

Firstly, the national health research systems analysis (HRSA) data can be obtained through review of existing HRSA reports. Where such data does not exist, it can be obtained through an assessment of national health research systems using HRSA toolkit [65-67]. Secondly, the health knowledge management systems (HKMS) data may be available in existing HKMS reports, and thus, a review of such reports might suffice. In countries where such reports do not exist, it will be necessary to conduct an assessment of HKMS using the "Research Matters" Knowledge Translation Toolkit [68]. Thirdly, a review of existing NHIS reports may avail information needed in HDGI. However, if those reports do not exist, it may be necessary to do an assessment of NHIS using the Health Metrics Network tool [69]. Lastly, the data for assessing ICT connectivity can be collected from health workers at various levels of national health system through use of the questionnaire mentioned earlier to gather information on dissemination of NHP and NHSP.

#### Ethical practises in health research and service provision

Data on dissemination of international ethical guidelines for medical practise and health research, bioethics review system, and institutionalization of ethics training should be collated simultaneously with that on HRSA (mentioned above) and using the same toolkit [65]. The module entitled 'Module 7000: Research ethics and ethical processes' is specifically designed for this purpose.

#### Macroeconomic and political stability

Firstly, the linkage between NEDP, PRSP and NHP/NHSP can be ascertained through a review of those documents. Secondly, a review of Medium-Term Expenditure Framework (MTEF) document will help to determine whether it contains a clear health component derived from NHSP. Finally, a review of the national constitution and in-depth interview with chairperson of the national legal bar association can facilitate identification of the extent to which a non-violent process exists by which those in authority are elected and replaced. This information can be complemented with a review of data on the Economist Intelligence Unit Democracy Index [40] and the Ibrahim Index of African governance [41].

## Summary

The weak governance and leadership in health development might explain why many countries in Africa are not on track to attain the health MDGs by 2015 [16,70,71]. This paper has attempted to review briefly existing governance frameworks and has proposed a modified framework on health development governance with a Health Development Governance Index. It has also suggested possible sources of data for estimating HDGI. The individual health development governance sub-functions indices can aid policy-makers to locate the sources of inadequate governance and then to develop appropriate interventions for ameliorating the situation.

One of the possible reasons for inadequate governance and leadership in health development in Africa is largely because many health leaders and managers, at various levels of national health systems, were never trained to govern and lead. Thus, whereas they may have had very good training on disease prevention and control, their curricula might not have featured training on governance and leadership. Therefore, there may be need to revise the curricula of schools of public health, medical schools, nursing schools and other schools of health sciences in Africa to reflect the recent developments in health systems performance assessment, including leadership and governance. In addition, leadership and governance should feature prominently in the continuing education programmes for medical and public health practitioners which are organized by the national professional associations (e.g. medical and nursing associations).

## List of abbreviations

The list of abbreviations include: AE: Allocative efficiency; CTP: Household's capacity to pay; DHS: Demographic and Health Surveys; GAVI: The Global Alliance for Vaccines and Immunization; GFATM: Global Fund to Fight AIDS, Tuberculosis and Malaria; HDG: Health development governance; HDGI: Health Development Governance Index; HDGSFI -Health development governance sub-function index; HE: Household expenditure on health; HFC: Health fairness in financial contribution; HKMS: Health knowledge management systems; HRSA: National health research systems analysis; ICT: Information, communication and technology; LR: Leadership Responsibilities index; LSMS: Living Standards Measurement Study; MDGs: Millennium Development Goals; MICS: Multiple Indicator Cluster Surveys; MTEF: Medium-term expenditure framework; NEDP: National economic development plan; NHIS: National health information system; NHP: National health policy; NHSP: National Health Strategic Plan; PEFA: Public Expenditure and Financial Accountability; PRSP: Poverty Reduction Strategy Paper; SC: Steering committee; TE: Technical efficiency; TWG: Technical Working Group; UNDP: United Nations Development Programme; WHO/AFRO: World Health Organization Regional Office for Africa; and WHS: World Health Surveys.

## Competing interests

The authors declare that they have no competing interests.

## Authors' contributions

JMK and DGK contributed equally to the design, analysis and writing of various sections of the manuscript. Both authors read and approved the final manuscript.

## Author details

JMK has a PhD in economics (health economics specialization) from the University of York, UK; MA and BEd in Economics from the University of Nairobi, Kenya; and a Diploma in health economics from the University of Tromso, Norway. DGK holds a MPH from the London School of Hygiene and Tropical Medicine, UK; and a PhD in Public Health from the University of New South Wales, Australia. Currently, JMK works at the World Health Organization, Regional Office for Africa, B.P. 06, Brazzaville, Congo. DGK is CDC/WHO Consultant, P.O. Box 529, Freetown, Sierra Leone.

## References

[B1] Kirigia JM The essence of leadership in health development African Journal of Health Sciences 2008 15 1-2 1 3

[B2] WHO World Health Report 2000 - Health systems: improving performance. Geneva 2000

[B3] HarperCollins Publishers Collins Pocket Dictionary & Thesaurus. London 2003

[B4] United Nations Development Programme Governance of sustainable human development: a UNDP Policy Document. New York 1997

[B5] Kaufman D Kraay A Zoido-Lobaton P Governance matters 1999 Policy Research Working Paper No. 2196. Washington, D.C.: The World Bank

[B6] World Health Organization Strengthening health systems to improve health outcomes: WHO's framework for action. Geneva 2007

[B7] Siddiqi S Masud TI Nishtar S Peters DH Sabri B Bile KM Jama MA Framework for assessing governance of the health system in developing countries: gateway to good governance 2008 Cairo: World health Organization Regional Office for Eastern Mediterranean 10.1016/j.healthpol.2008.08.005 18838188

[B8] Heifetz RA Laurie DL The work of leadership Harvard Business Review 2001 5 14 10174450

[B9] Kotter JP What leaders really do Harvard Business Review 2001 103 111 10104518

[B10] WHO/AFRO Guidelines for developing national health policies and plans. Brazzaville 2005

[B11] High Level Forum Paris declaration on aid effectiveness: ownership, harmonization, alignment, results and mutual accountability 2005 Paris: OECD

[B12] WHO/AFRO Addis Ababa Declaration on Community Health 2006 Brazzaville: WHO

[B13] WHO/AFRO Ouagadougou Declaration on Primary Health Care and Health Systems in Africa: achieving better health for Africa in the New Millennium. Brazzaville 2008

[B14] Murray CJL Frenk J A framework for assessing the performance of health systems Bulletin of the World Health Organization 2000 78 6 717 731 10916909 PMC2560787

[B15] Commission on Social Determinants of Health Achieving health equity: from root causes to fair outcomes 2008 Geneva: World Health Organization

[B16] International Finance Corporation The business of health in Africa: partnering with the private sector to improve people's lives 2008 Washington, DC: The World Bank

[B17] Kirigia DG Beyond needs-based health funding: resource allocation and equity at the state and area health service levels in New South Wales - Australia 2010 Doctor of Philosophy Thesis. Sydney: University of New South Wales http://unsworks.unsw.edu.au/vital/access/manager/Repository/unsworks: 8032-(2010)

[B18] McIntyre D Mooney G The economics of health equity 2007 London: Cambridge University Press

[B19] Murray CJL Knaul F Musgrove P Xu K Kawabata K Defining and measuring fairness in financial contribution to the health system 2000 GPE Discussion Paper Series. No. 24. Geneva: World Health Organization

[B20] Whitehead M The concepts and principles of equity and health 1991 Copenhagen: World Health Organization Regional Office for Europe

[B21] Masiye F Kirigia JM Emrouznejad A Sambo LG Mounkaila A Chimfwembe D Okello D Efficient management of health centres human resources in Zambia Journal of Medical Systems 2006 30 473 481 10.1007/s10916-006-9032-1 17233160

[B22] Osei D George M d'Almeida S Kirigia JM Mensah AO Kainyu LH Technical efficiency of public district hospitals and health centres in Ghana: a pilot study Cost Effectiveness and Resource Allocation 2005 3 9 10.1186/1478-7547-3-9 16188021 PMC1253524

[B23] Renner A Kirigia JM Zere AE Barry SP Kirigia DG Kamara C Muthuri HK Technical efficiency of peripheral health units in Pujehun district of Sierra Leone: a DEA application BMC Health Services Research 2005 5 77 10.1186/1472-6963-5-77 16354299 PMC1334185

[B24] Kirigia JM Emrouznejad A Sambo LG Munguti N Liambila W Using Data Envelopment Analysis to measure the technical efficiency of public health centers in Kenya Journal of Medical Systems 2004 28 2 155 166 10.1023/B:JOMS.0000023298.31972.c9 15195846

[B25] Kirigia JM Emrouznejad A Sambo LG Measurement of technical efficiency of public hospitals in Kenya: Using Data Envelopment Analysis Journal of Medical Systems 2002 26 1 39 45 10.1023/A:1013090804067 11777310

[B26] Zere EA Addison T McIntyre D Hospital efficiency in Sub-Saharan Africa: evidence from South Africa South African Journal of Economics 2001 69 2 336 358 10.1111/j.1813-6982.2001.tb00016.x

[B27] Kirigia JM Lambo E Sambo LG Are public hospitals in Kwazulu-Natal province of South Africa Technically Efficient? African Journal of Health Sciences 2000 7 3-4 25 32 17650022

[B28] Kirigia JM Emrouznejad A Cassoma B Asbu EZ Barry S A performance assessment method for hospitals: the case of Municipal Hospitals in Angola Journal of Medical Systems 2008 32 6 509 519 10.1007/s10916-008-9157-5 19058655

[B29] Kirigia JM Emrouznejad A Vaz RG Bastiene H Padayachy J A comparative assessment of performance and productivity of health centers in Seychelles International Journal of Productivity & Performance Management 2008 57 1 72 92

[B30] Kirigia JM Asbu Z Greene W Emrouznejad A Technical efficiency, efficiency change, technical progress and productivity growth in the national health systems of continental African countries Eastern Africa Social Science Research Review 2007 23 2 19 40 10.1353/eas.2007.0008

[B31] Kirigia JM Ovberedjo M Challenges facing National Health Research Systems in the WHO African Region African Journal of Health Sciences 2007 14 3-4 100 103

[B32] Kirigia JM Wambebe C Status of national health research systems in ten countries of the WHO African Region BMC Health Services Research 2006 6 135 10.1186/1472-6963-6-135 17052326 PMC1622748

[B33] Landry R Amara N Pablos-Mendes A Shademani R Gold I The knowledge-value chain: a conceptual framework for knowledge translation in health Bulletin of the World Health Organization 2006 84 597 602 10.2471/BLT.06.031724 16917645 PMC2627427

[B34] WHO A framework for country health information system development, Health Metrics Network. Geneva 2006

[B35] Kirigia JM Seddoh A Gatwiri D Muthuri LHK Seddoh J E-health: Determinants, opportunities, challenges and the way forward for countries in the WHO African Region BMC Public Health 2005 5 137 10.1186/1471-2458-5-137 16364186 PMC1327685

[B36] Council for International Organizations of Medical Sciences (CIOMS) International Ethical Guidelines for Biomedical Research Involving Human Subjects. Geneva 2002 14983848

[B37] Kirigia JM Wambebe C Baba-Moussa A Status of national research bioethics committees in the WHO African region BMC Medical Ethics 2005 6 10 10.1186/1472-6939-6-10 PMC1274319 16242014

[B38] Kirigia JM Muthuri LK Kirigia DG Health-related biotechnology transfer to Africa: principal-agent relationship issues African Journal of Medicine and Medical Sciences 2007 36 suppl 81 90 17703570

[B39] Houerou P Taliercio R Medium Term Expenditure Framework: from concept to practice. Preliminary lessons from Africa 2002 Africa Region Working Paper Series No. 28. Washington, DC: The World Bank

[B40] The Economist Intelligence Unit Limited The Democracy Index 2010. London 2010 http://www.eiu.com/democracy

[B41] Mo Ibrahim Foundation 2010 Ibrahim Index of African governance. London 2010 http://www.moibrahimfoundation.org/en/section/the-ibrahim-index

[B42] United Nations Development Programme Human development report 2003: Millennium Development Goals: a compact among nations to end human poverty 2003 New York: Oxford University Press

[B43] WHO World Health Surveys database http://www.who.int/healthinfo/survey/en/index.html Accessed on 9 March 2011

[B44] World Bank Public Expenditure and Financial Accountability Secretariat Website http://www.pefa.org/Accessed on 8 March 2011

[B45] PEFA Secretariat Public Financial Management Performance Measurement Framework 2006 Washington, D.C.: The World Bank

[B46] Zere E Moeti M Kirigia JM Mwase T Kataika E Equity in health and healthcare in Malawi: analysis of trends BMC Public Health 2007 7 78 10.1186/1471-2458-7-78 17504530 PMC1884146

[B47] Zere E Tumusiime P Walker O Kirigia JM Mwikisa C Mbeeli T Inequities in utilization of maternal health interventions in Namibia: implications for progress towards MDG 5 targets International Journal for Equity in Health 2010 9 16 10.1186/1475-9276-9-16 20540793 PMC2898738

[B48] O'Donnell O van Doorslaer E Wagstaff A Lindelow M Analyzing health equity using household survey data: a guide to techniques and their implementation 2008 Washington, D.C.: The World Bank

[B49] World Bank Living Standards Measurement Study (LSMS) http://www.worldbank.org/lsms/Accessed on 7 March 2011

[B50] ICF Macro Demographic and Health Surveys (DHS) http://www.measuredhs.com/Accessed on 7 March 2011

[B51] UNICEF Multiple Indicator Cluster Surveys (MICS) http://www.childinfo.org/index2.htm Accessed on 7 March 2011

[B52] World Bank ADePT-Health http://www.worldbank.org/adept

[B53] Kirigia JM Mensah OA Mwikisa CN Asbu EZ Emrouznejad A Makoudode P Hounnankan A Technical efficiency of zone hospitals in Benin The African Health Monitor 2010 12 30 39

[B54] Marschall P Flessa S Assessing the Efficiency of Rural Health Centres in Burkina Faso: An Application of Data Envelopment Analysis Journal of Public Health 2009 17 87 95 10.1007/s10389-008-0225-6

[B55] Sebastian M Lemma H Efficiency of the Health Extension Programme in Tigray, Ethiopia: A Data Envelopment Analysis BMC International Health and Human Rights 2010 10 16 10.1186/1472-698X-10-16 20546597 PMC2893104

[B56] Akazili J Adjuik M Jehu-Appiah C Zere E Using Data Envelopment Analysis to Measure the Extent of Technical Efficiency of Public Health Centres in Ghana BMC International Health and Human Rights 2008 8 11 10.1186/1472-698X-8-11 19021906 PMC2605432

[B57] Zere E Mbeeli T Shangula K Mandlhate C Mutirua K Tjivambi B Kapenambili W Technical efficiency of district hospitals: Evidence from Namibia using data envelopment analysis Cost Effectiveness and Resource Allocation 2006 4 5 10.1186/1478-7547-4-5 16566818 PMC1524815

[B58] Kirigia JM Sambo LG Scheel H Technical efficiency of public clinics in Kwazulu-Natal province of South Africa East African Medical Journal 2001 78 2 S1 S13 12002061 10.4314/eamj.v78i3.9070

[B59] Kibambe J Koch S DEA Applied to a Gauteng Sample of Public Hospitals South African Journal of Economics 2007 75 351 368 10.1111/j.1813-6982.2007.00125.x

[B60] Masiye F Investigating Health System Performance: An Application of Data Envelopment Analysis to Zambian Hospitals BMC Health services research 2007 7 58 10.1186/1472-6963-7-58 17459153 PMC1878476

[B61] Akazili M Adjuik M Chatio S Kanyomse E Hodgson A Aikins M Gyapong J What are the technical and allocative efficiencies of public health centres in Ghana? Ghana Medical Journal 2008 42 4 149 155 19452023 PMC2673839

[B62] Tlotlego N Nonvignon J Sambo LG Asbu EZ Kirigia JM Assessment of productivity of hospitals in Botswana: A DEA application International Archives of Medicine 2010 3 27 10.1186/1755-7682-3-27 21054835 PMC2992505

[B63] World Health Organization, Regional Office for Africa Hospitals economic efficiency analysis data collection instrument. Brazzaville 2000

[B64] World Health Organization, Regional Office for Africa Health centres economic efficiency analysis data collection instrument. Brazzaville 2000

[B65] WHO Health Research System Analysis Network Health Research Systems Analysis (HRSA) Toolkit. Geneva 2008 http://www.tropika.net/svc/specials/hrsa-toolkit/pages/components

[B66] D'Souza C Sadana R Why do case studies on national health research systems matter? Identifying common challenges in low- and middle-income countries Social Science and Medicine 2006 62 8 2072 2078 16165261 10.1016/j.socscimed.2005.08.022

[B67] Pang T Sadana R Hanney S Bhutta ZA Hyder AA Simon J Knowledge for better health: A conceptual framework and foundation for health research systems Bulletin of the World Health Organization 2003 82 11 815 820 PMC2572351 14758408

[B68] International Development Research Centre (IDRC) and the Swiss Agency for Development and Cooperation (SDC) The RM Knowledge Translation Toolkit: A Resource for Researchers 2008 Toronto: IDRC http://www.tropika.net/svc/specials/KT-Toolkit/pages/KT-Toolkit

[B69] Health metrics network Assessing the National Health Information System: An Assessment Tool (version 4) 2008 Geneva: WHO http://www.who.int/healthmetrics/tools/en/

[B70] Cavagnero E Daelmans B Gupta N Scherpbier R Shankar A Assessment of the health system and policy environment as critical complement to tracking interventions coverage for maternal, newborn, and child health The Lancet 2008 371 1284 93 10.1016/S0140-6736(08)60563-2 18406863

[B71] United Nations The Millennium Development Goals Report 2010. New York 2010 http://www.un.org/millenniumgoals/reports.shtml

